# Low Temperature Plasma Nitriding of Inner Surfaces in Stainless Steel Mini-/Micro-Pipes and Nozzles

**DOI:** 10.3390/mi8050157

**Published:** 2017-05-13

**Authors:** Tatsuhiko Aizawa, Kenji Wasa

**Affiliations:** 1 Department of Engineering and Design, Shibaura Institute of Technology, 3-19-10 Shibaura, Minato-City, Tokyo 108-8548, Japan; 2 Research and Development Division, TECDIA, Co., Ltd., 4-3-4 Shibaura, Minato-City, Tokyo 108-0023, Japan; k_wasa@tecdia.co.jp

**Keywords:** micro-fabrication, mini- and micro-nozzles, stainless steels, plasma nitriding, inner surfaces, hollow cathode device, nitrogen interstitials, hardness

## Abstract

Metallic miniature products have been highlighted as mini-/micro-structural components working as a precise mechanism, in dispensing systems, and in medical operations. In particular, the essential mechanical parts such as pipes and nozzles have strength and hardness sufficient for ejecting viscous liquids, solders, and particles. A low-temperature plasma nitriding process was proposed as a surface treatment to improve the engineering durability of stainless steel mini-/micro-pipes and nozzles. Various analyses were performed to describe the inner nitriding process only, from the inner surface of pipes and nozzles to their depth in thickness. AISI316 pipes and AISI316/AISI304 nozzle specimens were used to demonstrate by plasma nitriding for 14.4 ks at 693 K that their inner surfaces had a hardness higher than 800 HV.

## 1. Introduction

Pipes and nozzles are typical mechanical parts for transporting gaseous and liquid media and depositing liquid- or melt-drops onto material surfaces and interfaces in joining, soldering, and drawing processes [1]. In cellular phones, many devices and sensors are joined onto liquid crystal panels by adhesives, which are deposited onto the joined interface [2]. For example, digital camera units are integrated into cellular phones by accurately joining a series of functional parts. Dimensional accuracy and integrity is preserved by joining via adhesive droplets. These nozzle outlets contact and hit the joined surfaces; the stainless steel nozzles often deform and become damaged in the joining process. The ruby nozzle is often selected for sufficient hardness in operation.

High-density plasma nitriding has been used for hardening metals and alloys such as stainless steels, tool steels, and titanium and aluminum alloys with the use of DC-biased metallic tubes (referred to here as hollow-cathode devices) [3,4,5,6]. In these processes, these metallic alloy parts and components are efficiently plasma-nitrided in hollow-cathode devices. For example, AISI-420 martensitic stainless steel die-parts have been plasma-nitrided at 673 K for 14.4 ks to have a hardness higher than 1000 HV [3,7,8]. In addition, AISI304 and AISI316 stainless steel plates were also plasma-nitrided at 673 K for 14.4 ks by 70 Pa to have a nitrided layer thickness higher than 80 µm and a hardness higher than 1400 HV, respectively [9,10,11].

In the present study, this surface treatment, with the use of a hollow-cathode device, was employed to harden the inner surfaces of mini-/micro-nozzles and pipes for the joining process. The inner diameter of their through-holes ranged from 50 µm to 10 mm. The hydrogen–nitrogen mixed gas was blown into the inlet of these nozzles. The nitrided through-hole surfaces were observed by Scanning Electron Microscopy (SEM), and their hardness was measured via micro-Vickers testing. In the case of the micro-nozzles with a through-hole diameter of 50 µm, their inner surface hardness increased up to 950 HV on average after plasma nitriding at 693 K for 14.4 ks.

## 2. Experimental Procedure

### 2.1. RF-DC Plasma Generation System

**Figure 1:**
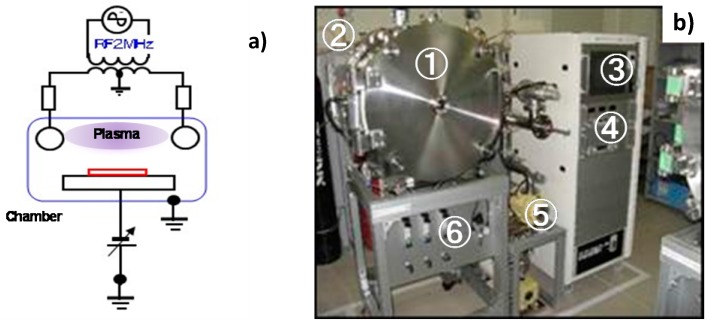
RF-DC plasma process system. (**a**) Illustration of system. (**b**) Developed plasma nitriding system in this study.

The low-temperature plasma nitriding process with use of the RF-DC plasmas had several superior features to the conventional processes [12,13]. The lower holding temperature with shorter duration time was cost-effective, and, the nitrided surface was free from roughing with less nitride precipitates. Since the inner surface roughening was disliked for nozzles, the present nitriding process with nitrogen super-saturation can afford to harden without significant roughing as suggested by [14]. Figure 1a illustrated the RF-DC plasma nitriding system. The dipole electrodes worked to ignite the RF-plasma, which was attracted to the DC-biased plate. Since the vacuum chamber was electrically neutral, the above RF-field as well as DC-biased field were arbitrarily placed and controlled independently in the chamber. This concept of plasma generation and control was put into practice. In Figure 1b, this RF-DC plasma nitriding system mainly consisted of six sectors: (1) vacuum chamber; (2) RF-generator; (3) control panel; (4) power generator; (5) evacuation units; and, (6) carrier gas supply.

### 2.2. Hollow Cathode Device

**Figure 2:**
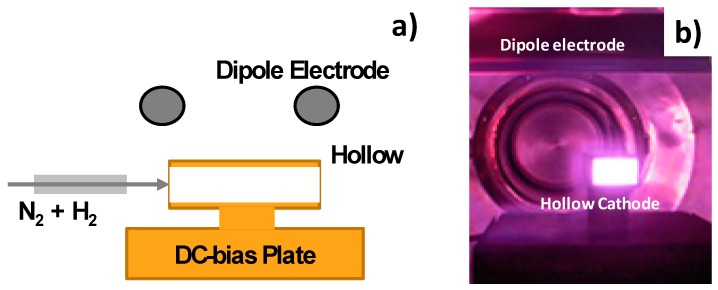
The hollow cathode device. (**a**) Illustration of the hollow cathode device to ignite the nitrogen plasmas in the inside of nozzles. (**b**) High densification in the hollow cathode.

In order to ignite the nitrogen plasmas only in the nozzle through-holes as well as the pipes, the RF-plasmas were confined in the inside of electrically conductive space. The hollow cathode effect to increase the ion and electron densities in the hollow helped to design how to accommodate the high-density nitrogen plasmas selectively in the through hole. Figure 2a illustrated a typical hollow cathode setup. The mixed carrier gas of nitrogen and hydrogen with the specified flow rate ratio was blown into the inlet of hollow tube through the flexible metal-bellows. 

**Figure 3:**
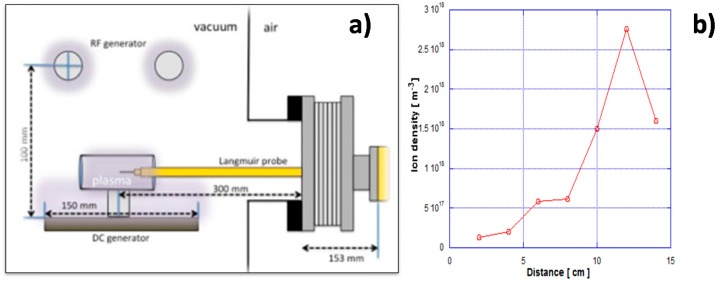
Langmuir probe measurement in the inside of hollow. (**a**) Experimental setup for measurement. (**b**) Ion density distribution from the inlet to the outlet.

The hollow tube was DC-biased so that the nitrogen/hydrogen RF plasma was confined in this through-hole; the ionization of nitrogen and hydrogen gases should take place only in its inside. In the preliminary studies [15], {N*, N^+^, NH (or NHx)} were detected by Emissive-light Optical Spectroscopy (EOS) and used to optimize the nitriding conditions. After three dimensional electromagnetic analyses in [16], the electrons are confined in the hollow by the electromagnetic shield at the outlet to enhance the ionization process. As shown in Figure 2b, the emissive light from the inside of hollow was intensified in the experiments with the RF voltage of 250 V and the DC-bias of −500 V. Figure 3 depicted a typical ion density distribution, measured by the Langmuir probe from the inlet toward the outlet of hollow with the length of 120 mm [16]. Since the ionization was enhanced by the carrier gas flow, the nitrogen ion density increased monotonically toward the outlet of hollow. This high density abruptly decreased down in one or two orders just at the outside of this hollow. This ion density distribution was preferable to make selective nitriding of the through holes; in particular, the inner surface of nozzle outlet could be efficiently nitrided. After the Langmuir probe measurement and emissive-light optical spectroscopy, the species of activated nitrogen atoms and NH*x*-radicals might be responsible for nitriding process of the austenitic stainless steel pipes and nozzles as discussed in [17]. As suggested in [18], the flow rate ratio of H_2_ to N_2_ in the mixture gas also affected the NH-radical yields in the generated plasmas. The nitriding condition with the RF voltage of 250 V, the DC-bias of −500 V, the pressure of 70 Pa and the N_2_/H_2_ ratio by 160 mL/min to 30 mL/min was used as standard processing parameters in the following experiments. The holding temperature was measured by the thermocouple, which was embedded in the jig to fix the pipe and nozzle on the cathode plate. The heating unit was on/off controlled to keep the measured temperature by 693 K.

### 2.3. Specimen

Besides for the normal AISI316 (Tokai Engineering Service Co. Ltd., Tokyo, Japan) pipe unit with the inner diameter of 16.9 mm, both the AISI316 mini- and AISI304 micro-nozzles were prepared to make plasma nitriding with use of the hollow cathode device. Table 1 lists their dimensional sizes and geometries, respectively. The finished inner surfaces by mechanical machining and end-milling was nitrided for pipe and nozzle specimens only after cleaning. The AISI316 austenitic stainless steel pipe was first used to describe the hollow cathode effect on the hardening behavior along its inner surface. Both the mini- and micro-nozzles were also utilized to selectively harden the through-hole surfaces. Scanning Electron Microscopy (SEM) was utilized to describe the nitrided layer in their inner surfaces.

## 3. Experimental Results

### 3.1. Plasma Nitriding of AISI316 Stainless Steel Pipe

In a manner similar to the hollow cathode device in Figure 2a, the nitrogen ion density is expected to increase along the carrier gas flow direction through the pipe and nozzle since the nitrogen atom and NH–radical flux intensity is enhanced toward the outlet of the pipe and nozzle. That is, the amount of nitrogen interstitial atoms into the depth of the pipe and nozzle thickness is expected to increase from their inlet to the outlet. First, AISI316 austenitic stainless steel pipe is employed to investigate this hollow cathode effect on the nitriding behavior. With an increase in ion density, the nitriding process is enhanced by increasing the nitrogen atoms and NH–radical flux into the depth of the pipe thickness. This enhancement results in a high hardness.

**Figure 4:**
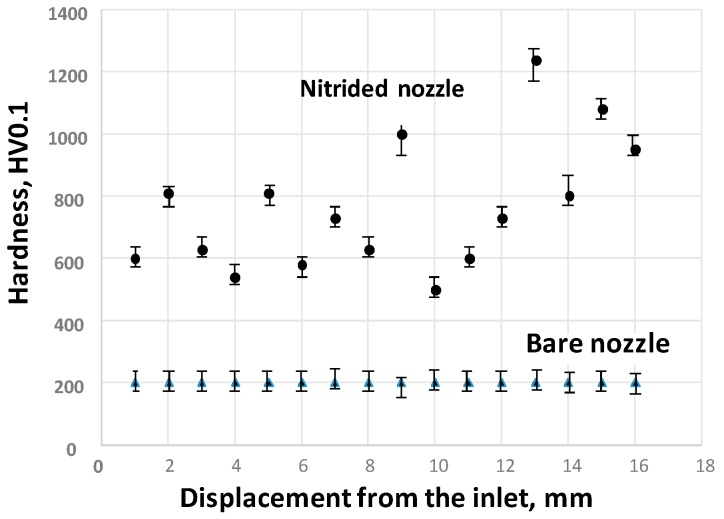
Comparison of the measured hardness distribution toward the end of the outlet in the normal nozzle through-hole before and after the plasma nitriding process.

Figure 4 compares the measured hardness distributions from the inlet to the outlet of the AISI316 pipe before and after plasma nitriding at 693 K for 14.4 ks by 70 Pa. A specimen with a length of 16.9 mm was cut off from the end of the outlet in this pipe after nitriding. The average hardness of nitrided through-hole inner surfaces is 800 HV higher than the matrix hardness of 200 HV at every position. 

In correspondence with the monotonic increase of ion density in Figure 3b, the measured hardness also increases toward the outlet of the nozzle. This implies that a through-hole of the pipe works as a hollow cathode device, and only its inner surface is selectively nitrided.

### 3.2. Plasma Nitriding of AISI304 Mini-Pipe

**Figure 5:**
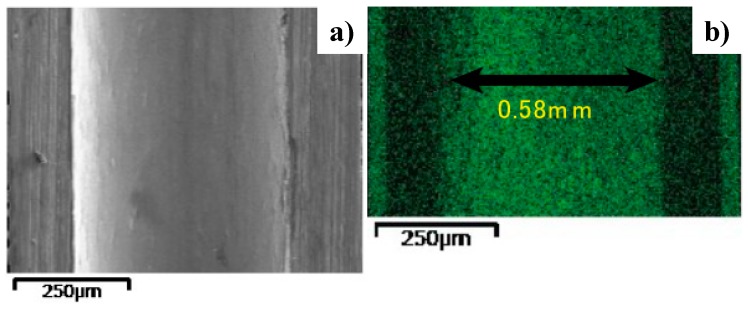
The plasma-nitrided AISI304 mini-pipe. (**a**) Scanning Electron Microscopy (SEM) image of the inner surface of nitrided pipe. (**b**) Nitrogen mapping on the inner surface.

The AISI304 mini-pipe specimen was employed to make SEM and Energy Dispersive Spectroscopy (EDX) analyses on the microstructure of plasma-nitrided inner surfaces. The nitriding condition was the same as that used in the case of the AISI316 pipe. The nitrided mini-pipe at 673 K for 7.2 ks by 70 Pa was cut in half; the specimen for analysis was prepared by mechanical and chemical polishing. Figure 5 depicted the SEM image and nitrogen mapping by EDX, respectively. The surface condition did not change significantly from the initial smooth surface with a roughness around 0.5 µm. The nitrogen was uniformly distributed on the inner surface of the pipe; the nitrogen content became around 5 mass %. This means that the inner surface of the pipes should be nitrided to have a nitrogen content value that is higher than the maximum nitrogen solubility limit of 0.1 mass %.

### 3.3. Plasma Nitriding of the Mini-Nozzle

**Figure 6:**
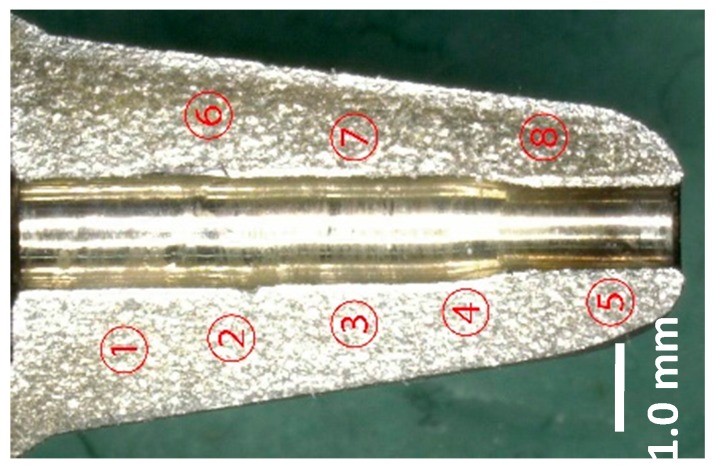
Outlet of mini-nozzle after plasma nitriding at 693 K for 14.4 ks.

The mini-nozzle in Table 1 is widely utilized as a guide structure to control the movement of the fibers. The hardening of its inner surface is essential to prolong its lifetime. This mini-nozzle has a through-hole, the inner diameter of which decreases to 1.3 mm at the outlet. After plasma nitriding at 693 K for 14.4 ks, the nitrided mini-nozzle was wire-cut in half via electric discharge machining. The micro-Vickers testing was performed with a diamond indenter. Figure 6 depicts the optical microscope image with the hardness measurement locations from #1 to #8 along the nozzle outlet.

As listed in Table 2, the hardness at #2 and #6 away from the through-hole inner surface is lower than 400 HV; this might be because the plasma density becomes lower than that at the vicinity of the outlet of the nozzle. In fact, the hardness values at #1 and #2 differ from each other even when both points are located closer; the inhomogeneous nitriding behavior must be attributed to a lower plasma density.

On the other hand, the hardness, measured at the inner surface area of the nozzle outlet, reached 600–800 HV. This proves that the inner surface of the through-hole in the mini-nozzle is nitrided and hardened at a significant depth. 

**Figure 7:**
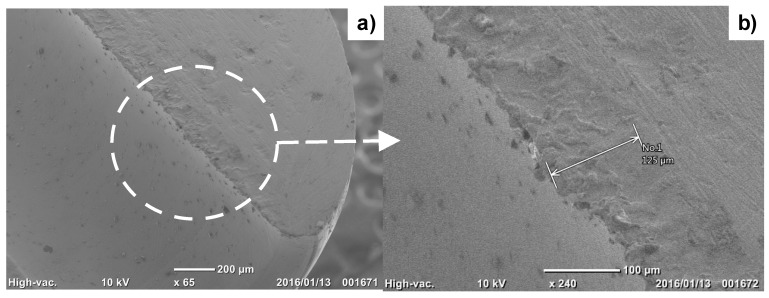
SEM image of the cross section for the outlet mini-nozzle thickness. (**a**) The nitrided layer is formed along the through-hole in the low magnification SEM image. (**b**) Its thickness is estimated to be 125 µm in the high magnification SEM image.

Figure 7 shows the SEM image of a cross section of the mini-nozzle. Based on a noticeable difference in microstructure, the affected layer thickness by this nitriding was estimated to be 125 µm. 

Based on Table 2, a hardness higher than 600 HV was measured at #3–5 and #7–8, in the vicinity of the top of mini-nozzle outlet. This implies that the inner surface of the through-hole of the mini-nozzle was plasma-nitrided to the extent that a hardness much higher than 200 HV for the bare AISI316 stainless steels was attained.

### 3.4. Plasma Nitriding of Micro-Nozzle

The micro-nozzle, which was frequently used to join electric parts, had a thin and narrow through-hole, the inner diameter of which was 200 µm at the outlet. Since the metallic melts, the solders, and the chemically active agents flow along the inside, its inner surface must be hardened and chemically modified. 

**Figure 8:**
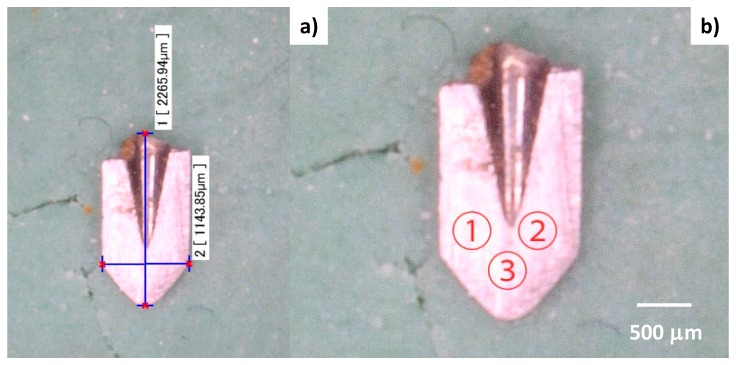
A cross section of the plasma nitride micro-nozzle at 693 K for 14.4 ks. (**a**) Hardness testing specimen cut-off from the original micro-nozzle. (**b**) The hardness measuring points from #1 to #3 on the flattened surface in the specimen.

In the nitriding process, the specially designed jig was prepared to fix this micro-nozzle so that the mixed gas flow direction coincides with the center line of the through-hole of the micro-nozzle. In a manner similar to the post-treatment of the mini-nozzle, the specimen for analysis was prepared from the plasma-nitrided micro-nozzle. Figure 8 depicts the optical microscopic image of the specimen with hardness measurement locations from #1 to #3 at the vicinity of the nozzle outlet inner surface. The positions of #1 and #2 are located at the vicinity of the inner surface of the through-hole, while #3 is selected to locate only on the inner surface of the through-hole.

Table 3 summarizes the measured micro-Vickers hardness at #1 to #3. Compared to the hardness of bare AISI316, 200 HV, the inner surface of the through-hole was significantly hardened. The average hardness for this micro-nozzle was 950 HV, much higher than that for the mini-nozzle. This is because the micro-indenter included more un-nitrided area at the outlet of a mini-nozzle to lower the measured hardness in practice. On the other hand, in the case of this micro-nozzle, the nitrided area of its thickness at the outlet mainly contributes to the measured hardness.

**Figure 9:**
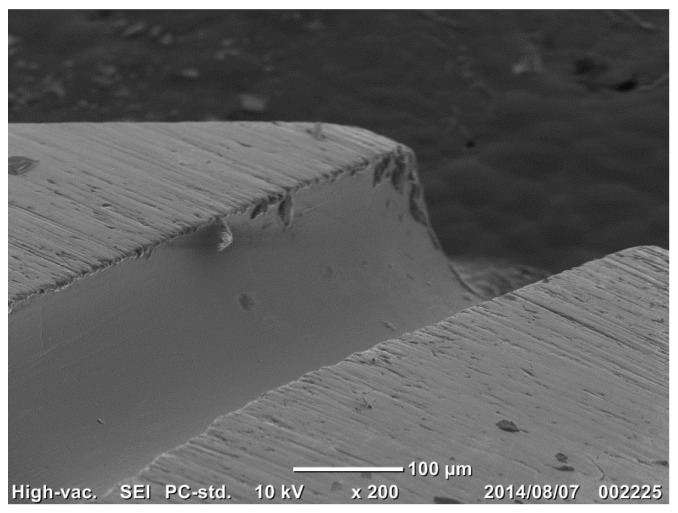
SEM image of a cross section at the outlet of the micro-nozzle with an inner diameter of 200 µm.

Figure 9 shows the SEM image of a cross section of the outlet of the through-hole. Although the fine nitriding front end was not observed in the thickness, the deeper nitrided layer might be formed by the present hollow cathode-type plasma nitriding.

## 4. Discussion

Several surface modification processes are thought to improve the mechanical and chemical properties of through-hole surfaces in stainless steel pipes and nozzles. It is difficult to blow the resource gases into the through holes of pipes and nozzles for the hard coating and to stimulate the physical or chemical deposition processes. With respect to case hardening and the nitriding in gaseous and liquid phases, the entire surfaces were affected by these processes; in addition, little modification took place on the inner surfaces. In normal plasma nitriding of austenitic stainless steels, a holding temperature higher than 800 K was necessary, even for nitride precipitation hardening. That formation of precipitates has a possible risk of deteriorating the inner surface quality of through-holes in miniature nozzles.

In the present high-density, low-temperature nitriding, the inner surface of nozzles and pipes is selectively nitrided by the hollow-cathode effect. As shown in Figure 7 and Figure 9, the nitrided layer thickness reaches to 1/8 to 1/4 of the micro-nozzle thickness. Just as reported in [8,19], the nitrided layers with thicknesses around 75–80 µm were formed by the RF/DC and RF plasma nitriding at 673 K. In particular, the highly densified plasma by the hollow-cathode effect provides a means of forming a thick nitrided layer at the inner surface of the narrow channels in the mini- and micro-nozzles. In addition, as recently pointed out in [8,11], the nitrided layer in the stainless steel matrix has a refined microstructure with an average grain size of less than 0.1 µm. This implies that the nitrided micro-nozzle has a composite structure where its inner part is nitrided to have a hardness of up to 1000 HV and high strength, and the other outer sections in the thickness remains a bare stainless steel with its intrinsic ductility and toughness. This composite structure has hardness–toughness balancing for preserving the integrity of micro-nozzles to make flushing out the viscous solders and polymer melts from its outlet to achieve joining and drawing.

Toward the industrial application of this technology, the fixture for introducing the mixture gas into the micro-nozzle needs to be first redesigned to nitride the tens or hundreds of micro-nozzles simultaneously. As keenly stated in [18], the distributed nitriding system using the hollow cathode in the cascade provides a means of performing simultaneous nitriding. Each nitriding unit in the cascaded system works as a miniature nitriding reactor with a substantial reduction of processing time in heating and cooling. Mock-up testing has recently begun as part of a METI program to nitride 36 micro-nozzles by a single process.

## 5. Conclusions

Low-temperature high-density plasma nitriding was proposed to make surface modifications to mini- and micro-nozzles. The inner surface of through-holes in these nozzles was sufficiently hardened, creating a hardness higher than that of the bare AISI316 by three to five times. This solution can be applied to improve the strength and hardness of manifold nozzles and to replace ceramic outlet nozzles. Furthermore, this surface modification is also capable of strengthening fine channels and miniature reservoirs in Micro Electro Mechanical Systems (MEMS).

## Figures and Tables

**Table 1:** Geometry, size and dimension of pipe, mini- and micro-nozzles to be processed by the present plasma nitriding.

Pipe and Nozzle	Length	Outer Diameter at Outlet	Thickness at Outlet	Inner Diameter at Outlet	Surface Roughness Rz	Outlook	Material Supplier
AISI304 Micro-Pipe	30.0 mm	0.88 mm	0.15 mm	0.58 mm	0.5 μm	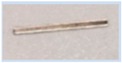	TECDIA Co., Ltd.
AISI316 Pipe	55.5 mm	21.5 mm	2.3 mm	16.9 mm	1.5 μm	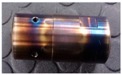	Tokai Engineering Service, Co., Ltd.
AISI316 Mini-Nozzle	25.0 mm	2.8 mm	0.86 mm	1.08 mm	1.2 μm	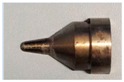	Tokai Engineering Service, Co., Ltd.
AISI304 Micro-Nozzle	5.5 mm	1.9 mm	0.5 mm	1.0 mm	0.5 μm	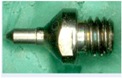	TECDIA Co., Ltd.

**Table 2:** Hardness distribution at the vicinity of the inner surface of the through-hole in the mini-nozzle outlet.

Position in Figure 4	Micro-Vickers Hardness
#1	530 HV ± 15 HV
#2	390 HV ± 20 HV
#3	660 HV ± 10 HV
#4	750 HV ± 15 HV
#5	820 HV ± 20 HV
#6	380 HV ± 10 HV
#7	690 HV ± 20 HV
#8	600 HV ± 15 HV

**Table 3:** Hardness distribution at the vicinity of the inner surface of the through-hole in the micro-nozzle outlet.

Position in Figure 6	Micro-Vickers Hardness
#1	960 HV ± 30 HV
#2	880 HV ± 20 HV
#3	1020 HV ± 15 HV
